# Algorithms for Recollection of Search Terms Based on the Wikipedia Category Structure

**DOI:** 10.1155/2014/454868

**Published:** 2014-01-29

**Authors:** Stijn Vandamme, Filip De Turck

**Affiliations:** Department of Information Technology (INTEC), Ghent University-iMinds, Gaston Crommenlaan 8, Bus 201, 9050 Gent, Belgium

## Abstract

The common user interface for a search engine consists of a text field where the user can enter queries consisting of one or more keywords. Keyword query based search engines work well when the users have a clear vision what they are looking for and are capable of articulating their query using the same terms as indexed. For our multimedia database containing 202,868 items with text descriptions, we supplement such a search engine with a category-based interface whose category structure is tailored to the content of the database. This facilitates browsing and offers the users the possibility to look for named entities, even if they forgot their names. We demonstrate that this approach allows users who fail to recollect the name of named entities to retrieve data with little effort. In all our experiments, it takes 1 query on a category and on average 2.49 clicks, compared to 5.68 queries on the database's traditional text search engine for a 68.3% success probability or 6.01 queries when the user also turns to Google, for a 97.1% success probability.

## 1. Introduction

Search engines today are predominantly “keyword query” based: a user enters a query consisting of one or more keywords, and the search engine returns an ordered list of documents that contain the words and are about the concept the keywords denote. This works very well when the user knows the common name for the concept he is looking for.

Other information retrieval systems allow browsing through category labels and suggestions. This is more helpful in the use case where the user just cannot remember the word he is looking for at the moment or in the use case where the user has no clear or only a moderate end-goal or definition of his information need. In the following two subsections, we discuss these two use cases. Subsequently, the contribution and the outline of this paper are described.

### 1.1. Tip-of-the-Tongue Experience

People occasionally fail to retrieve a word or a name from memory, often combined with partial recall and the feeling that retrieval is imminent: the word or name is colloquially said to be “on the tip of one's tongue.”

Faced with such a tip-of-the-tongue experience, the user now actually faces *two* related information retrieval problems, each with a specific end goal. First, the user wants to recollect the name or search term he fails to remember at the moment, which is referred to as the “*recollection problem.*” Second, he wants to find the documents about that search term. That was his original information retrieval problem.

A dataset may not contain all the associations and categories that could help the user to recollect the word or name. In this paper, the dataset is a multimedia database where each fragment has a text description. Such a description will include the name of the people in the fragment, but not necessarily their functions, categories they belong to, or other biographical details. To solve the recollection problem, finding the word or the name, a text-based search engine on the dataset itself is of little use. A search for the word or the name in a different dataset such as Wikipedia or the Internet, where more information is available, is often more effective.

The disadvantage of searching in a different dataset is that the set of described concepts is different than the set of concepts in the original dataset and likely much larger. This is especially true for Wikipedia or the entire Internet.

### 1.2. Browsing and Surfing

Datta et al. [1] classify search sessions based on user intent and clarity of the end-goal. When the user who is looking for items about a subject and is very clear about the specific subject he is looking for, the user's behavior is called *searching*. When such a user is capable of naming that subject, the traditional text-based search engine is very useful and effective. The user enters that subject as a query, and the result set includes all the items whose description includes the query term(s). Typically, such a search session can be short.

When the user has a moderate clarity of the end-goal, the user's behavior is called *surfing*. When the user has no clear end-goal, it is called *browsing*. In a surfing or browsing session, using a traditional search engine requires the user to sequentially devise and enter different queries, which may or may not be related to each other.

The possibility to navigate through a category graph by following links relieves the surfing user from having to come up with and type the terms for successive related queries, which is normal behaviour for users of a traditional search engine. Boldi et al. [2] classifies this behaviour, called query reformulation, in generalization, specialization, error corrections, and parallel moves. With the addition of a category structure to browse through, our system facilitates category generalization and specialization.

### 1.3. Contribution

In this paper, we present a search system for a multimedia database with textual annotations that allows the user to browse a category structure, tailored to the content of the database. The category structure is taken from Wikipedia, but concepts that do not appear in the original dataset are pruned, that is, removed from the category structure. We demonstrate that, faced with a tip-of-the-tongue experience, this addition allows the user to search for named entities in a limited number of clicks, smaller than the number of queries he would otherwise have to formulate. We measure the distribution of the numbers of categories shown and demonstrate it to be helpful for surfing.

### 1.4. Outline

In Section 3, we present our system, Wikipedia's category structure on which our system relies and the multimedia database on which we implemented the system. In Section 4, we explain the details of the algorithm to match words and phrases with Wikipedia's articles and categories, the algorithm to tune Wikipedia's category structure to the contents of the database, and the user interface. In Section 5, we evaluate our system by measuring the distribution of the fan-out of the pruned category structure and the effort to resolve an information retrieval when the user experiences a tip-of-the-tongue experience and is unable to name the named entity he is searching for.

## 2. Related Work

A browsing or surfing experience can be improved by displaying suggestions. Even before the rise of the search engines, Lieberman [3] already presented the Letizia system that made recommendations to assist browsing. The graph from which Letizia made its suggestions was not a category tree but a link tree: the web page that the user visited, the documents to which that starting page linked, the documents that they link to, and so on. The Letizia system selected in this graph of linked pages those documents with terms that interest the user, based on the terms that appeared often in his browsing history. Hassan-Montero and Herrero-Solana [4] use tags from folksonomies, displayed in a tag-cloud, to guide the navigation of a user's browsing or searching experience. Unlike the category structure, folksonomies do not have hierarchical structure.

Lots of research is devoted to generating good recommendations, in information retrieval contexts as well as in other contexts, such as recommendations for music, movies, and restaurants. Zhang et al. [5] and Pazzani and Billsus [6] give an overview of tag-aware and content-based recommendation systems.

For searching large collections of similar-style items, user interfaces that allow searching based on facets were developed [7]. An early faceted search prototype was Flamenco [8]. Ben-Yitzhak et al. [9] developed a more sophisticated hierarchically faceted search implementation in the Lucene search library. A conceptual difference with this work is that facet-based search engines typically allow searching on the properties of the items themselves (from images to products in a web shop), rather than the properties of the named entities mentioned in the documents of the search space, as is the case in our system.

The category structure in Wikipedia, on which our work is based, has been used for a wide range of purposes. Vercoustre et al. [10] use Wikipedia's category structure for entity ranking, where articles are considered relevant if the concept is associated with the right category, among other criteria. Kaptein et al. [11] use the same technique to improve the results of ad hoc retrieval, where there is no categorical restriction. Schönhofen [12] uses Wikipedia categories for identifying document topics. Nastase and Strube [13] use the category structure to acquire knowledge, with special attention on subcategories based on traits. Zesch and Gurevych [14] use the category graph for natural language applications, such as determining semantic relatedness. Yu et al. [15] explore the properties of Wikipedia's categories and use them for evaluating ontologies. Demartini et al. [16] use Wikipedia's category structure as one of several data sources for finding named entities in Wikipedia. Pehcevski et al. [17] use categories and the locality of links in Wikipedia for entity ranking. Hahn et al. [18] bring faceted search to Wikipedia, allowing search for concepts with their own Wikipedia pages, based on their properties when available in Wikipedia in the form of structured information. We use the category structure instead to facilitate browsing multimedia items about the entities of a given category and, furthermore, demonstrate the usefulness of our system when searching for multimedia items on a named entity in case of a tip-of-the-tongue experience.

Medelyan et al. [19] provide an extensive overview of research that mines Wikipedia information, using Wikipedia, its category structure, redirects, disambiguations, page links, infoboxes, and templates, as an encyclopedia, as a corpus, as a thesaurus, as a database, as an ontology, as a network structure, or for a variety of other purposes.

## 3. Functional Overview

**Figure 1:**
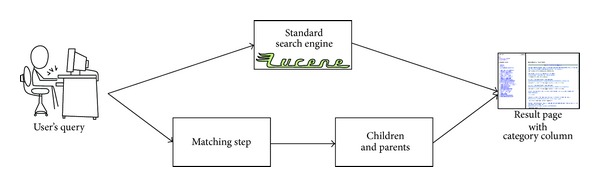
Generation of a result page at query time.

Our system is based on a traditional text-based search engine on the multimedia database. In response to a query request, the system generates a result page. This page contains the same results as the result page generated by a standard search engine. Our system adds a left column to the user interface, which allows browsing through the pruned category graph, when the query matches a nonpruned Wikipedia article and/or category.  Figure 1 illustrates the generation of a result page in response to a query. If the query matches a Wikipedia article, the left side of the result page lists the nonpruned categories containing the article; if a query matches a nonpruned Wikipedia category, it lists both the category's containing parent categories and the category's nonpruned articles and subcategories.

These two sides of the result page are generated in parallel: the system looks up if there is a branch of the category structure matching the query, and if so, it displays the direct ascendants and descendants.

**Figure 2:**
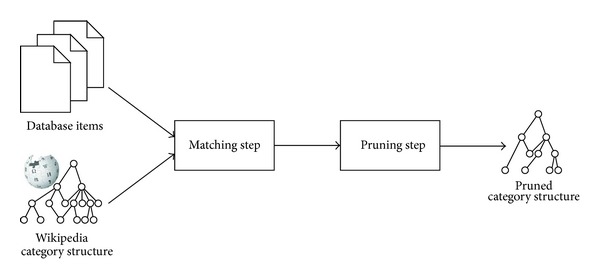
Off-line generation of the pruned category structure.

Our system is able to generate the left column since it stores a modified version of Wikipedia's category structure. The modification consists of two steps, as illustrated in Figure 2: concepts not covered by the database's content are removed from the category structure. All categories whose descendant articles are not covered by the database's content are removed as well. Categories containing only one article on a subject covered by the database's content are removed, and the single remaining subject is placed directly under the category's parent(s). This latter step is referred to as the pruning step.

This modified version of Wikipedia's category structure is generated once, offline. It needs to be regenerated when the database's content changes and can also be regenerated when Wikipedia and its category structure evolves.

### 3.1. Wikipedia's Category Structure

Wikipedia [20] is a free, web-based, collaborative, and multilingual encyclopedia project. It has editions in hundreds of languages; for 104 languages the Wikipedia edition contains over 100,000 articles. Wikipedia categorizes its articles in categories: each article in Wikipedia is included in zero or more categories; it is a stated Wikipedia policy that each article should be included in at least one category. In general, categories represent major topics and their main use within Wikipedia is in finding useful information. Categories can also include subcategories. In this article, we exclude the categories in the Wikipedia project namespace, whose names start with the prefix “Wikipedia:.” Those categories are used for housekeeping purposes.

Excluding those categories in the Wikipedia project namespace, there are two types of categories: topic categories or set categories. A topic category, where the topic is usually expressed in a singular word or name, contains articles relating to the subject. For example, the category “Biology” contains articles in the field of biology and the category “France” contains articles relating to the topic France. By contrast, a set category is named after a class, usually in plural form, and contains the articles about the elements in that class. For example, the category “Cities in France” contains articles whose subjects are cities in France. There are a limited number of categories that belong to both types, called set-and-topic categories. An example is as “Voivodeships of Poland,” which contains articles about particular voivodeships as well as articles relating to voivodeships in general.

It is common for categories with many descendants to be subdivided into subcategory by a trait of the subjects. For example, the category “Musical group” has the subcategories “Musical group by genre” and “Musical group by nationality”: the first contains subcategories such as “Dance musical groups,” “Folk musical groups,” “Rock musical groups,” etc.; the second contains subcategories such as “French musical groups,” “German musical groups,” and so forth. Especially the subdivision of large categories “by nationality” or “by country” is very common.

The Wikipedia category system is a directed acyclic graph, where the nodes are the Wikipedia articles and categories and the directed edges indicate the “is parent of” relationship. It is possible for a category to be a subcategory of more than one parent, but a category can never be a descendant of itself. The “descendants” of a category are the articles it contains, the subcategories it contains, and, recursively, the descendants of its subcategories.

There is a unique top-level category “Contents,” of which all categories are descendants, possibly via multiple paths.

An article and a topic category may share the same name. Such categories are called eponymous categories. For example, most countries and major towns have both an article dedicated to them and a category with lots of related articles. Generally, the eponymous category contains the article with the same name.

In this paper, we work with Wikipedia's category system stricto sensu. Articles consisting only of links to instances of some concept are not part of the category system we use. Semantically, there is little semantic difference between a link in the article “List of Asian Countries” and an article about a country being included in the set category “Countries in Asia” [21], but the category system stricto sensu is readily available structured information whereas the semantic meaning of a link being an item in a list is implicit and possibly ambiguous.

### 3.2. The Multimedia Database

For this paper, we work with a database that contains broadcasted television material from Flemish public and commercial broadcasters. At the moment of writing, the database contains about 15,000 hours of broadcasted television material, for the most part news. This corresponds to 202,868 individually annotated television items.

Each item is manually annotated with both keywords from a dictionary and a “free text” description. 63,000 different keywords are used in tags and 209,500 different words used in the description. Most text is in Dutch. Additional metadata for each multimedia item includes the programme title and the date on which the item was broadcasted. In total, the textual metadata comprises 440 MiB.

### 3.3. Implementation Choices

The traditional text-based search engine is powered by Lucene [22]. The engine indexes (after stemming) the textual metadata.

Since the textual descriptions of the multimedia database are mostly in Dutch, we use the Dutch language version of Wikipedia. This edition contains 855,590 articles and 49,810 categories.

The system is written in Java, as a Tomcat web application, running on an Apache web server usage. The system is deployed on a host with 2 quad-Core AMD Opteron processors 2350 and 8 GiB RAM.

The category graph is generated by a MySQL procedure and stored in a MySQL database.

## 4. Algorithm Details

### 4.1. Matching Wikipedia's Articles and Categories

In the textual metadata, words and phrases appear that denote concepts covered by Wikipedia articles and categories. Yet, the textual metadata in the database does not contain all the concepts covered by all Wikipedia articles and categories.

**Figure 3:**
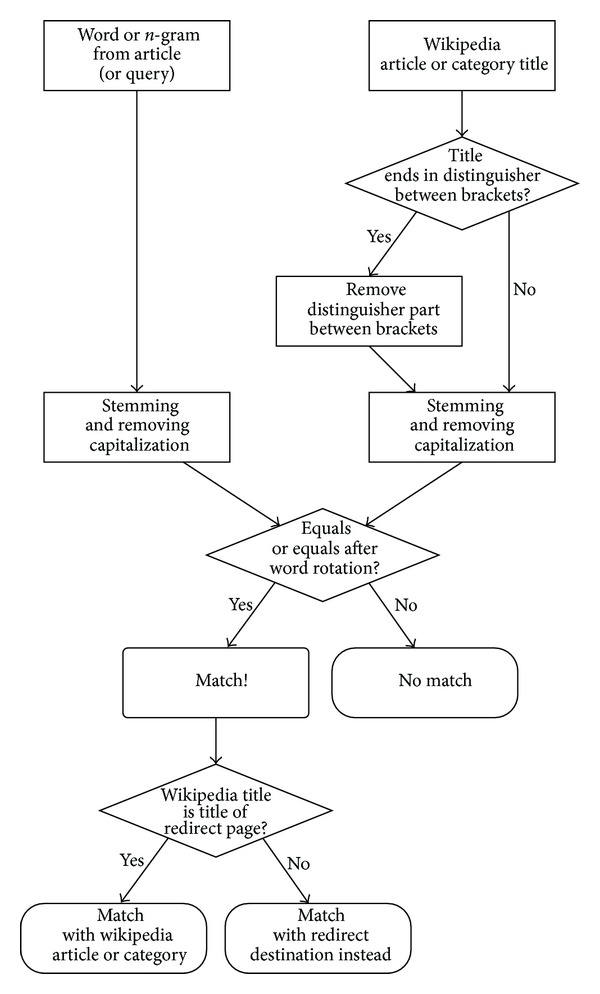
Flowchart of the matching algorithm.

To determine whether a Wikipedia article or category deals with a subject that is covered by a database item, we determine if the textual metadata contains the article's title or the title of a redirect page that redirects to the article. The algorithm to determine whether a snippet of text in the metadata matches a Wikipedia article or category is represented in Figure 3. We apply stemming both to the text and the title of the Wikipedia article. We also match names with Wikipedia articles when the title is a rotation of the name as it appears in the metadata; that is, “van het Groenewoud Raymond” will match the Wikipedia article “Raymond van het Groenewoud,” even if no redirect article exists.

Thanks to Wikipedia's redirection mechanism, when synonyms of the titles of an article appear in the textual metadata, they can also be matched to the Wikipedia concept if a redirect page between the synonym and the article exists.

When a term is the title of more than one Wikipedia article, whose titles only differ because of distinguishing suffix between brackets, such as the articles “Namur (city)” and “Namur (province),” we match the term with all of these articles. When a term in the textual metadata matches an article that is a disambiguation page, we match with all the pages the disambiguation page directs to.

### 4.2. Pruning Wikipedia's Category Structure

The Wikipedia category system is a directed acyclic graph, where the nodes are the Wikipedia articles and categories and the directed edges indicate the “is parent of” relationship.

To optimize the category structure for use in our database, we prune Wikipedia's category structure by leaving out the categories that are not covered by the database items, and do not have any descendant dealing with a subject covered by the database items.

This pruning takes place offline, not at query time. Only when the contents of the database change, the part of the category structure that is needed needs to be updated as well.

**Figure 4:**
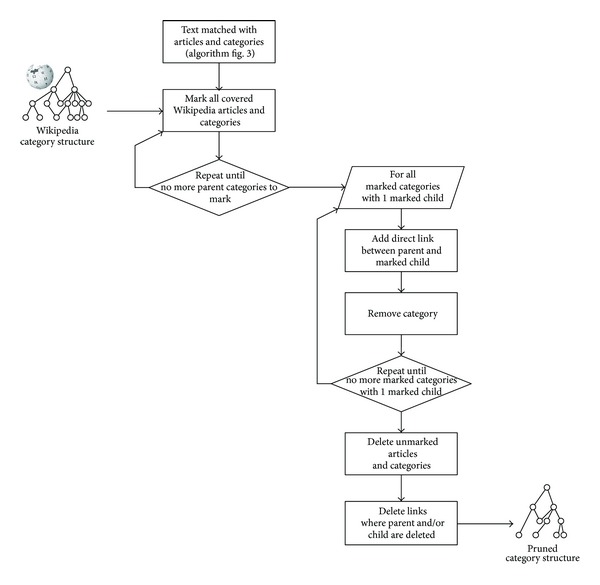
Flowchart for generating the pruned category structure.

The algorithm to generate the pruned category structure is represented in Figure 4. We replace iteratively the categories that are not covered by the database items and only contain a single article on a subject that is covered by the database items, with its only such member.

This procedure reduces the size of the category graph in a database-specific way. 86% of the Dutch language Wikipedia articles and categories are pruned; 122,804 out of 891,537 articles and categories remain, as detailed in Table 1. Eponymous categories and their similarly named articles are counted only once.

For example, in the Dutch version of Wikipedia, there are 15 articles and 2 subcategories in the category “Cities in Iceland.” The first category is “Capital of an Icelandic region,” which groups the capitals of the Icelandic regions. The other category is Reykjavik, a city with enough content about it in the Dutch Wikipedia to merit its own eponymous category. Since very few of the multimedia items in the database deal with Iceland, the pruned list is noticeably shorter. It consists of only the eponymous category Reykjavik and the city Reykjavik. The category “Capital of an Icelandic region” is pruned away since no capital of an Icelandic region appears in the database.

The pruned list for “Cities in Denmark” contains the city “Aalborg,” even though the category “Cities in Denmark” does not contain the article “Aalborg” directly. Instead, “Aalborg” is the title of an article in the category “Capital of a Danish region,” which is a subcategory of “Cities in Denmark.” But since no capital of a Danish region is mentioned in the database besides Aalborg, the category “Capital of a Danish region” is pruned away and Aalborg is lumped in directly in the category “Cities in Denmark,” together with the 7 Danish cities that are mentioned in the database and are not regional capitals.

This algorithm generates the pruned category structure in MySQL. We imported Wikipedia's page, categorylinks, and redirect tables in a MySQL database and implemented this algorithm in a MySQL procedure.

### 4.3. The User Interface

**Figure 5:**
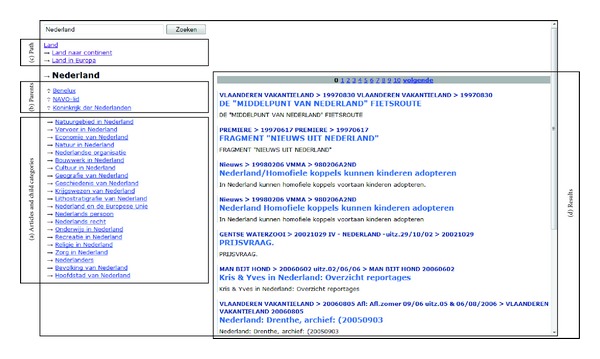
The user interface when the user entered “Land” (country) and subsequently clicked “Land naar continent” (country by continent), “Land in Europa” (Country in Europe), and “Nederland” (the Netherlands). The following areas of the user interface are marked (a) the articles and child categories of the category “Nederland,” (b) the parent categories of “Nederland,” other than “Land in Europa,” the one that is listed in (c), (c) the traversed categories, only when a link in (a) is followed, and (d)  results for searching the database with the query “Nederland,” as in the traditional search engine.

Like many other search engines, the user interface of our system includes a text field and a “Search” button, allowing the manual entry of a query. The rest of the user interface consists of two parts. The right side contains the results for the query just like the traditional search interface. Our system adds a left column, which displays a relevant part of the pruned category structure when the query matches a Wikipedia article or nonpruned category.  Figure 5 depicts the user interface.

When the query does not match a nonpruned Wikipedia concept, the left column of the screen is empty. This “relevant part of the category structure” consists of (a) the Wikipedia articles and child categories, when the query matches a Wikipedia category, and (b) the parent categories, when the query matches a Wikipedia article or nonpruned category.

When a link in (a) is followed, downwards in the category structure, the traversed path is kept visible in the top left corner. The last link in this path is necessarily a parent category of the concept identified by the clicked link. Since it already appears in (c), it is not repeated again in (b). When a link is clicked, both sides of the screen are updated.

The right side of the screen contains the results for the query, exactly as a traditional search engine responds. The output in this side of the screen is exactly the same when a link is followed, as when the text of the link is entered as a query in the input text field.

It is possible for a displayed link, indicating a nonpruned Wikipedia concept, to result in a screen without search results and therefore an empty right side of the screen, only displaying the notice “0 results.” This is very common for categories that have titles including traits, such as “Economist by nationality.” However, their list of articles and child categories (a) will always be nonempty, and the concepts in this list will always either result in a nonempty result set or have nonpruned descendants who have a nonempty result set.

## 5. Evaluation Details

### 5.1. Evaluation Methodology

First, we measure the distribution of the fan-out of the category structure and the pruned category structure, as we describe in Section 5.1.1. On our server, generating this pruned category structure takes 8 min 53 s to complete.

The distribution of the number of nonpruned articles and child categories among nonpruned categories gives us an indication of the length of options the user is presented while browsing.

Secondly, we measure the effort it takes, measured in number of queries entered and links clicked, to resolve an information retrieval problem when the user fails to recollect the name of named entity he is searching for. In Section 5.1.2 we explain how we determine this measure.

#### 5.1.1. Distribution of the Fan-Out of the Category Structure

We measure the distribution of the number of Wikipedia articles and child categories among all Wikipedia categories and among the Wikipedia categories that remain in the pruned category structure since they are covered by content in the multimedia database. Some of these articles and child categories may be pruned away in the pruned category graph. Therefore, we also measure the distribution of number of nonpruned articles and child categories among nonpruned categories. This is the distribution that is most relevant for the users of the system, since it is the distribution of the number of names they are presented with in the (a) section of the user interface when they enter or click a category.

#### 5.1.2. Effort to Find the Name of a Named Entity

To evaluate how such a pruned category structure helps in finding the right concepts, and eventually the right database items, we compare the effort a user has to make to satisfy the recollection problem. We compare the number of clicks in the interface the user has to make, starting from a broad category, to the number of attempts of discovering the concept by searching using related words as queries in the text-based search engine on the video database and in Google, with personalization of the results turned off [23]. That is because Google sometimes customizes search results based on past search activity on Google. We did not want our past search activity to influence the result page that Google serves us in answer to the queries posed in this experiment.

For the evaluation, we selected on 104 named entities: people, organizations, locations, and titles from 30 categories: singers, soccer teams, islands, music albums, and so forth. We only selected named entities that appear in at least one item in the database, therefore a query on their name never results in an empty result set. In the following subsections, we first describe how we determine the effort to find the name of a named entity without the category system and next how we measure the effort using the category system and make the comparison.

*(A) Characterization of the Effort to Find the Name of a Named Entity without the Category System.* When a user has to search for a named entity he cannot remember the name of, he can formulate related words as queries and hope that the name of the named entity appears in the result. In a true tip-of-the-tongue experience, the user recognizes the correct name as such once he stumbles on it. As such, we consider a single appearance of the name on the first result page to be an adequate resolution of the recollection problem. The search engine and Google are configured to display 10 results per page. The search engine outputs the fragment title plus one line of extra textual description per result fragment (as well as the programme name and broadcast date).

To select relevant related words for each named entity in this experiment, we turn once more to Wikipedia. According to Wikipedia's policy, the lead paragraph serves both as an introduction to the article and as a summary of its most important aspects. From this lead paragraph, we take the first 10 concepts, excluding text in parenthesis. For this purpose, we define concept as a noun or an adjective that can be turned into a noun through stemming (such as “French” to “France”). We treat these 10 concepts as the related concepts that are likely to come to mind to the user when thinking about the named entity, even as the user cannot recollect the named entity's name.

As evaluation, we find out if the names of these 104 named entities can be found by typing any such related concept as a query. We consider the named entity found when the result page displaying the first 10 result items, including their shortened descriptions, contains its name. If this is not the case for any of the 10 related concepts, we find out if the concept can be found by typing a combination of two such concepts as a query, with the same criterion used to determine a hit.

As an example, the first paragraph on Wikipedia for Harrison Ford is

 Harrison Ford (born July 13, 1942) is an American film actor and producer. He is famous for his performances as Han Solo in the original Star Wars trilogy and as the title character of the Indiana Jones film series.

From this paragraph, we take the related words “actor,” “producer,” “Han Solo,” “Star Wars,” and “Indiana Jones.” We use these related words as queries in Google and in our own standard search engine. When the result page with the first 10 results for the query consisting of the first related word does not contain the name we are looking for, we proceed with the second related word as query. When the result page for that query does not contain the name we are looking for, we proceed with the third related word and so on.

Invariably, there is at least one related word, like “actor” in Harrison Ford's case, indicating the category of the named entity among the first related words found like this.

In Google, Harrison Ford does not appear on the first result page for the queries “actor” or “producer,” and does appear when searching for “Han Solo.” In the textual search engine on the database, Harrison Ford does not appear on the first result page for the queries “actor,” “producer,” “Han Solo,” or “Star Wars,” but the name appears when the query is “Indiana Jones.”

The name we are looking for does not appear in all cases on the first result page for any of the first 10 related concepts that we took from the beginning of the Wikipedia text. After 10 attempts of single-related word queries we give up on single-related word queries and try a slightly different strategy. In such a case, we try the same procedure again with combinations of two of these related concepts as queries. We enter the first and the second related concept together as one query, then the first and the third, and so forth.

We measure the number of queries it takes until the result page includes the name of the named entity we are looking for. After this number of queries, the user's recollection problem is solved. The user then has to make one more query, entering the found named entity in the search box to get the results for the original information need.

*(B) Comparison with the Effort Using the Category System.* We compare the numbers of queries the user entered to find out the name of the named entity with the numbers of clicks the user has to make while browsing the category structure on our system. That is not an entirely fair comparison, as the effort of following a link is not the same as the effort of conjuring up a related concept and typing it. Note that the user clicks through the category structure after first entering one single-keyword query to begin with, such as “singer,” “soccer team,” “island,” and “music album,” followed by this number of clicks.

Note that after clicking the last of these links, our system will respond with the results for that named entity on the right side of the screen. As a consequence, the recollection problem is solved after the next-to-last of these clicks. With the last click, the user gets to the results of his original information retrieval problem.

For example, in the parsed category tree, it takes 1 query and 4 clicks from “actor” to “Harrison Ford”: the query “actor,” followed by clicks on “Actor by nationality,” “American actor,” “American film actor,” and “Harrison Ford.”

### 5.2. Evaluation Results

#### 5.2.1. Distribution of the Fan-Out of the Categories

**Figure 6:**
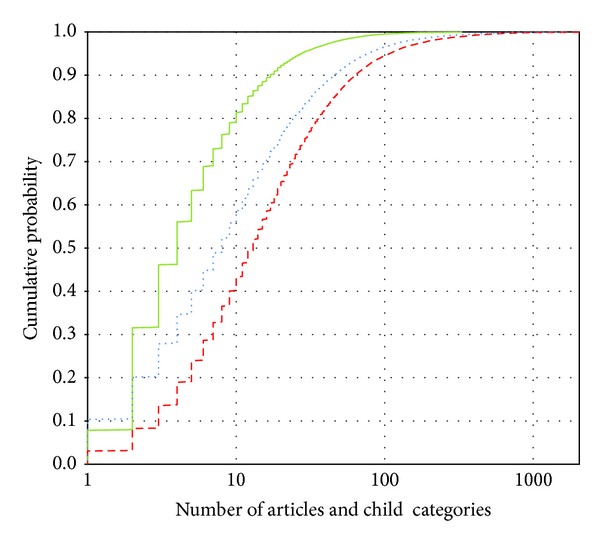
Cumulative distributions of the number of child articles and categories. Dashed: the number of child articles and categories in Wikipedia. Dotted:  the number of child articles and categories of nonpruned categories, for our multimedia database. Full line: the number of nonpruned children of nonpruned categories, for our multimedia database.

There are 49,810 categories in the Dutch language version of Wikipedia, excluding categories in the Wikipedia project namespace. The dotted line in Figure 6 plots the distribution of the numbers of articles and child categories. Eponymous categories and their similarly named article are counted only once. 147 categories (0.3%) have over 500 subcategories and articles. 73 of these categories list places or municipalities in specific countries, states, provinces, and so forth.

The dashed line in Figure 6 plots the distribution of the numbers of articles and child categories in the categories that are not pruned away. The average nonpruned category has more subcategories and member pages than the average Wikipedia category. This is not a surprise: the higher the number of member pages is, the higher the probability that two or more descendants will be mentioned in the database is. Of the 147 Wikipedia categories with over 500 subcategories and articles, 144 are not pruned away.

The distribution of the number of nonpruned articles and child categories in nonpruned Wikipedia categories is plotted in a full line. This is the distribution of the number of names the users are presented with in section (a) when they enter or click a category. Such a list is usually short enough not to overwhelm a user with the sheer amount of options.

For a good browsing experience, the user should not be overwhelmed by a long list of options but neither should he be required to follow too many links with only a couple of options to choose from each time.

In 79.9% of the cases, the user is presented with 10 or less subcategories and articles under a category. In 95.4% the pruned list is limited to 30 or less subcategories and articles. In Table 2, the distribution of non-pruned children in non-pruned categories is compared with the distribution of all children and of non-pruned children in all Wikipedia categories.

List (b) containing parent categories is even less likely to overwhelm the user. In the Dutch version of Wikipedia, all articles and categories have 23 or less parent categories; only 235 (less than 0.03%) have more than 10. In the pruned category structure, all matched concepts have 22 or less nonpruned parent categories; only 171 (0.6%) have more than 10.

The pruning of the category structure in a database-specific way eliminates the possibility of clicking on a subject that is not covered by the database and reduces the length of list (b) of articles and child categories considerably.

#### 5.2.2. Effort to Find the Name of a Named Entity

**Figure 7:**
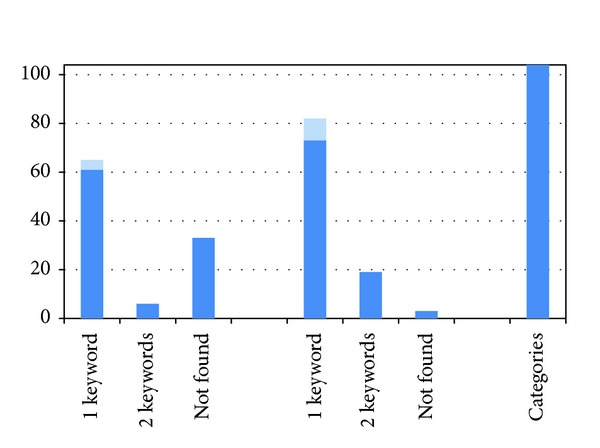
Number of named entities, out of 104, that were found using single related keyword queries and number that could not be found using single related keyword queries but was found using queries that contain 2 related keywords using the database's text search engine (left) and using Google (center). All named entities can be found clicking through the pruned category structure (right). Named entities found through queries with keywords that relate to specialized knowledge about the named entity are in lighter color.

In Figure 7 we present the number of named entities in our experiment where the name of the named entity appeared on the first result page with 10 results for any of the 10 related keywords and the number where the name did not come up for any of these 10 keywords, but where it did appear for a specific combination of 2 related keywords.

In a number of cases, the only related keyword that leads to the name of the named entity when entered as a query can only come to the user's mind if he is very knowledgeable about the subject, since the related keyword involves specialized knowledge about the subject. For example, a user that has a tip-of-the-tongue experience and cannot recollect the name of the former Belgian software company “Real Software” will find that name when he queries Wikipedia or Google with the NGO's founders Rudy Hageman and Leo Meuris, who are mentioned in the lead section. However, the names of these founders are not common knowledge, even among those who are reasonably knowledgeable about the former company. If the user does not know either of these founders' names, he will not find the company's name in the first 10 results in a 1-keyword or 2-keyword query (using related concepts as described above). In Figure 7, those cases are indicated in a lighter color.

**Figure 8:**
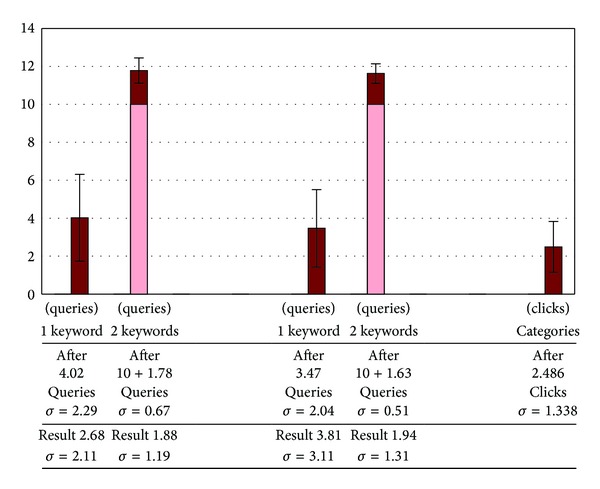
Number of queries actually typed before having a result including the named entity  among the first 10 results and, below, the ranking of the result that included the named entity, using the database's text search engine (left) and using Google (center), and numbers of clicks, after the first query, to find the name using our system (right).

In Figure 8, we compare how many queries we entered before we stumbled on the name we were looking for. We distinguish between the cases where the recollection problem was solved with single-keyword queries only and the cases where trying 10 different single-keyword queries did not provide results that included the name, but where double-keyword queries (displayed in Figure 8 in a lighter color) did.

When we drop the distinction, we find that it takes on average 4.68 queries to get to the result of the recollection problem in 68.3% of the cases using the database's search engine and on average 5.01 queries to get to the result of the recollection problem in 97.1% of the cases using Google.

It is no surprise that using Google for finding out the name of the named entity results more often in success, compared to using the database's traditional search engine. As mentioned in Section 1, the contents of the database describe the video fragments but do not provide much background information on the subject in those videos.

We compare the numbers of queries the user entered to find out the name of the named entity with the numbers of clicks the user has to make. That is not an entirely fair comparison, as the effort of following a link is not the same as the effort of conjuring up a related concept and typing it. Note that the user clicks through the category structure after first entering a single-keyword query to begin with, such as “singer,” “soccer team,” “island,” and “music album.”

**Figure 9:**
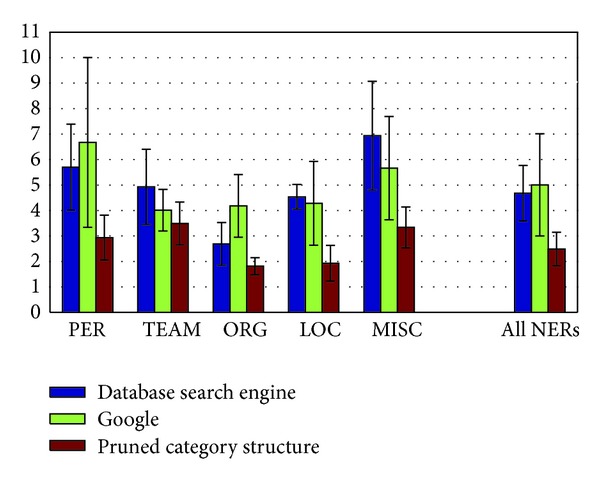
Number of queries actually typed before having a result including the named entity among the first 10 results, by type of named entity.

In Figure 9 we make the distinction between types of named entities: publicly known people, teams (including both sport teams and musical groups), organizations, locations, and miscellaneous named entities. This last category includes events, films, songs, and publication titles.

We notice from Figure 9 that the number of clicks it takes to find the name of the named entity is lower for organizations and locations than for people, teams, and miscellaneous named entities.

Among people and teams, the pattern of a subdivision of a category by trait, such as by nationality, is more common. In such cases, there is an intermediate category, therefore an additional click, between the general category and the descendant categories that group according to a given trait. In the Harrison Ford example, it took 2 clicks to go from “Actor” to “American actor.” The intermediate category was “Actor by nationality.”

Trying related keywords in the database's traditional search engine or in Google also requires more effort to find the names of people and miscellaneous named entities compared to organizations and locations. For sport teams and musical groups, the difference is less profound. This can be explained considering that such groups are more likely to have other named entities, often very specific to the groups, among the related words: a star player or coach for a sport team, a hit single for a music group.

Furthermore, all cases in our experiments where the strategy of trying keywords was unsuccessful in Google, even trying double-keyword queries, involved miscellaneous named entities, such as magazines, songs, and movies. The failure rate of this strategy in the database's text-based search engine was 2.25 times higher for miscellaneous named entities than the other types of named entities.

Yet, even when we only consider the cases where the search for miscellaneous named entities using the query reformulation strategy is successful, the effort to find the named entity and the database items mentioning them, using the pruned category system, is still 41.49% lower than reformulating queries in Google and 51.93% lower compared to reformulating queries in the database's search engine.

## 6. Conclusion

Wikipedia's category structure is helpful in a browsing or surfing experience, allowing the user to narrow or broaden the concept that he is querying. We implemented and presented an algorithm that prunes the category structure offline. The pruning is customized to the contents of a database. We used this pruned category structure in a system which allows querying that database for the categories mentioned in its items and browsing through these categories, presenting the user lists that are short enough to be helpful and not overwhelm him, yet complete enough for the information retrieval of all the information in the database.

In the use case of a user with a tip-of-the-tongue experience, unable to recollect the name of the named entity he is looking for, the system provides the user with logical and effective strategy, consisting of querying for a broader concept and then descending down the category structure. In our experiments, a user faced with a tip-of-the-tongue experience on a named entity mentioned in database items can find those items through 1 query for the category of the named entity and 2.49 clicks on average. This is significantly less effort than the 4.68 + 1 queries the user has to formulate and execute on the database's traditional text search engine for a 68.3% success probability or the 5.01 queries the user has to formulate on Google, followed by 1 additional query on the database's traditional text search engine, for a 97.1% success probability.

## Figures and Tables

**Table 1:** Number of articles and categories in the Dutch version of Wikipedia and in the pruned category structure.

	Dutch version of Wikipedia	Pruned category structure
Articles	855,590	101,477
Categories	49,810	28,144
Eponymous categories	13,863	6,817
Total	**891,537**	**122,804**

**Table 2:** Distribution of the number of child articles and categories.

Percentile	All children in Wikipedia categories	Nonpruned children in Wikipedia categories	Nonpruned children in pruned categories
25%	3	6	2
50%	8	13	4
75%	20	29	8
80%	24	36	10
85%	32	46	12
90%	46	64	17
95%	77	107	27
98%	142	209	49
99%	230	333	70
99.5%	363	484	98
